# The discovery of novel HDAC3 inhibitors via virtual screening and *in vitro* bioassay

**DOI:** 10.1080/14756366.2018.1437156

**Published:** 2018-02-21

**Authors:** Jie Xia, Huabin Hu, Wenjie Xue, Xiang Simon Wang, Song Wu

**Affiliations:** a State Key Laboratory of Bioactive Substance and Function of Natural Medicines, Department of New Drug Research and Development, Institute of Materia Medica, Chinese Academy of Medical Sciences and Peking Union Medical College, Beijing, China;; b Molecular Modeling and Drug Discovery Core Laboratory for District of Columbia Center for AIDS Research (DC CFAR), Department of Pharmaceutical Sciences, College of Pharmacy, Howard University, Washington, DC, USA

**Keywords:** Histone deacetylase 3, virtual screening, pose filter, *in vitro* bioassay, novel inhibitors

## Abstract

Histone deacetylase 3 (HDAC3) is a potential target for the treatment of human diseases such as cancers, diabetes, chronic inflammation and neurodegenerative diseases. Previously, we proposed a virtual screening (VS) pipeline named “Hypo1_FRED_SAHA-3” for the discovery of HDAC3 inhibitors (HDAC3Is) and had thoroughly validated it by theoretical calculations. In this study, we attempted to explore its practical utility in a large-scale VS campaign. To this end, we used the VS pipeline to hierarchically screen the Specs chemical library. In order to facilitate compound cherry-picking, we then developed a knowledge-based pose filter (PF) by using our in-house quantitative structure activity relationship- (QSAR-) modelling approach and coupled it with FRED and Autodock Vina. Afterward, we purchased and tested 11 diverse compounds for their HDAC3 inhibitory activity *in vitro*. The bioassay has identified compound **2** (Specs ID: AN-979/41971160) as a HDAC3I (IC_50_ = 6.1 μM), which proved the efficacy of our workflow. As a medicinal chemistry study, we performed a follow-up substructure search and identified two more hit compounds of the same chemical type, i.e. **2–1** (AQ-390/42122119, IC_50_ = 1.3 μM) and **2–2** (AN-329/43450111, IC_50_ = 12.5 μM). Based on the chemical structures and activities, we have demonstrated the essential role of the capping group in maintaining the activity for this class of HDAC3Is. In addition, we tested the hit compounds for their *in vitro* activities on other HDACs, including HDAC1, HDAC2, HDAC8, HDAC4 and HDAC6. We have identified these compounds are HDAC1/2/3 selective inhibitors, of which compound **2** show the best selectivity profile. Taken together, the present study is an experimental validation and an update to our earlier VS strategy. The identified hits could be used as starting structures for the development of highly potent and selective HDAC3Is.

## Graphical abstract

**Figure:**
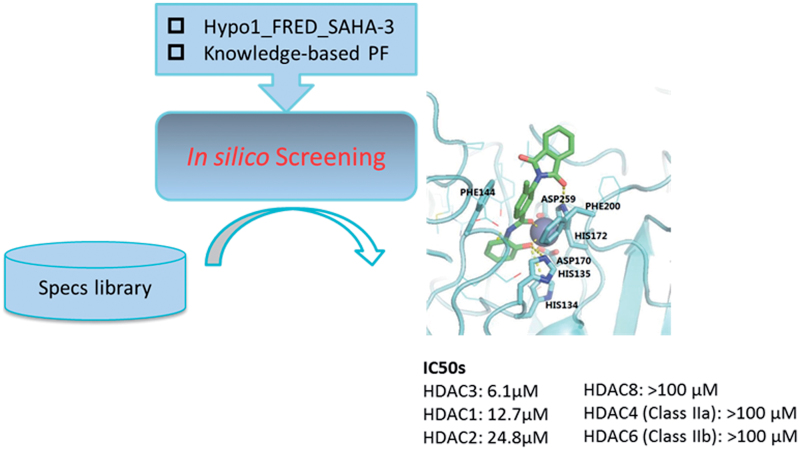

## Introduction

Histone deacetylase 3 (HDAC3) is a zinc-dependent enzyme and belongs to the HDACs family. Like the other members of this family, it functions to catalyse histone deacetylation thus is involved in the maintenance of acetylation homeostasis, i.e. a balance between acetylation and deacetylation^1^. Since the homeostasis of acetylation is critical to the precise regulation of gene transcription, functional abnormality of HDACs (e.g. HDAC3) or their binding partners may result in a range of human diseases^2–5^.

**Figure 1.**
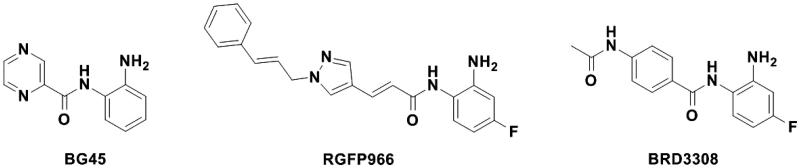
The reported HDAC3-selective inhibitors for the treatment of a range of diseases.

The role of HDACs in the development of cancer has been well studied^3^ ^,^ ^4^ ^,^ ^6^ ^,^ ^7^. Clearly, the functional upregulation or overexpression of HDACs causes the repression of gene transcription that regulates important cellular functions, and thus results in tumorigenesis^8^. Accordingly, the inhibition of HDACs is beneficial for cancer treatment. The FDA’s approval of HDACs inhibitors as anti-cancer therapy, i.e. vorinostat (or SAHA) and romidepsin (or FK228) is a strong proof for the druggability of HDACs as targets. Though currently the marketed drugs were pan-HDAC inhibitors, HDAC3 selective inhibitors were also used to treat multiple myeloma, e.g. BG45^3^ ^,^ ^9^. In addition to tumorigenesis, HDAC3 is uniquely linked to other diseases, mainly due to its role in regulating expression of specific genes in the pathology of corresponding diseases. Firstly, HDAC3 catalyses the deacetylation of nuclear factor κB (NF-κB), which further activates the inflammatory gene expression thus results in inflammatory responses^10–13^. Since cytokines-induced hyperactive inflammatory responses is the main pathology of chronic inflammatory diseases, e.g. chronic obstructive pulmonary disease (COPD), while HDAC3 inhibition is able to attenuate this disease process, HDAC3 has been considered as a potential target to discover drugs that treat chronic inflammatory diseases. Secondly, HDAC3 was identified as an important negative regulator of memory formation^14–17^. Earlier studies showed the focal deletion or the inhibition of HDAC3 may lead to the activation of gene transcription required for memory processes, e.g. nuclear receptor subfamily 4 group A member 2 and c-fos^14^. And it was recently identified that the deacetylase domain was the structural basis for HDAC3 to affect memory formation and this domain played distinct roles in specific brain regions^17^. HDAC3 is also associated with Huntington’s disease^18–21^, partly due to its involvement in the expression of macrophage migration inhibitory factor (MIF) associated with glial cell activation^22^. It was reported that HDAC3 inhibitors significantly reduced MIF levels thus led to reduced astrocyte activation in the N171-82Q transgenic mouse model^22^. This study demonstrated the potential of using HDAC3 inhibitors for the treatment of Huntington’s disease. Thirdly, growing evidences had uncovered the genetic association between HDACs and diabetes, and suggested HDACs as potential targets in diabetes^23–29^. Recently, HDAC3 was validated as the most critical isoform to affect pancreatic β-cell function^30^ ^,^ ^31^. The inhibition of HDAC3 alone *in vitro* and *in vivo* has proved the efficacy in treating both type 1 and type 2 diabetes^31–33^. Accordingly, HDAC3 inhibitors (HDAC3Is) could be used as a therapeutic approach for the above mentioned diseases. To the best of our knowledge, the reported HDAC3 selective inhibitors so far only include BG45 for cancer^9^, RGFP966 for neurological diseases^22^ ^,^ ^34^ and BRD3308 for diabetes^31–33^ (cf. Figure 1). In case that these HDAC3Is fail to enter the next stage of drug research and development pipeline due to currently unknown drawbacks, chemically dissimilar HDAC3Is are still needed.

For rapid identification of diverse hits, high-throughput virtual screening (VS) is undoubtedly a great option^35–37^. We have been working on the development of cheminformatics methods for more efficient VS against HDACs. One issue we focused on was the method to construct maximal unbiased benchmarking data sets (MUBD) for the evaluation of both ligand-based VS (LBVS) and structure-based VS (SBVS) approaches^38^. The application of our method to the superfamily of HDACs and Sirtuins led to the release of MUBD-HDACs^2^. Most recently, the subset for HDAC3, i.e. MUBD-HDAC3 facilitated the rational design of the VS strategy/pipeline, i.e. Hypo1_FRED_SAHA-3 for HDAC3Is discovery^39^. Another important issue to which we have been dedicated was the construction of target-specific pose filters (PFs)/classifiers, which aimed to replace the role of human experts in cherry-picking of compounds for bioassay by automatically inspecting binding modes. Our in-house methods used protein−ligand pairwise atomic maximal charge transfer potential based on Delaunay tessellation (PL/MCT-tess) descriptors to build QSAR-based models and have been applicable to the situation where either only one or multiple crystal structures of protein-ligand complexes are available^40^ ^,^ ^41^.

The prior efforts mentioned above established a good foundation for this study. In this work, we firstly performed a high-speed VS to retrieve potential inhibitors of HDAC3 from Specs chemical library via the use of the well-designed pipeline, i.e. Hypo1_FRED_SAHA-3. Then we used our in-house QSAR method to build a knowledge-based PF that could facilitate compound cherry-picking from the potential hits^40^. Since the application of PF required a pool of binding poses for each potential hit, we performed a new-round molecular docking to generate and keep multiple binding poses by using FRED and AutoDock Vina, respectively. Then we used the PF to automatically inspect the binding modes and recognize “native-like” poses. Based on the lowest Chemgauss4/Vina score of native-like binding poses, i.e. the score of the “native” pose, we selected top-ranking compounds from the lists of FRED and Vina, respectively. Eventually, we purchased and tested a few representative overlapping compounds so as to validate the practical utility of the PF optimized VS protocol. Based on the identified hit, we further performed substructure search in order to identify more potent hits of the same chemical class and gain the preliminary structure-activity relationship (SAR). In addition, we explored the selectivity profile of all the hit compounds by testing them against a panel of HDAC isoforms that attracted much interest, i.e. HDAC1/2/8 (class I), HDAC4 (class IIA) and HDAC6 (class IIB). This study represents our continued efforts towards the discovery of highly potent and selective HDAC3 inhibitors.

## Methods

### Chemical library and ligand preparation

The Specs chemical library (version Nov. 2015) that included approximately 210,000 compounds was downloaded from www.specs.net and used as the screening dataset. The “Prepare Ligands” module in Discovery Studio (DS) 2016 was utilized to prepare the chemical library. That module firstly generated all protonated states of each compound at the pH range of 7.3–7.5 and then enumerated all potential stereoisomers. Subsequently, all the protonated forms and stereoisomers, i.e. chemical entities that violated the Lipinski filters^42^, i.e. AlogP greater than 5, number of hydrogen bond donors greater than 5, number of hydrogen bond acceptors greater than 10 or molecular weight greater than 500, were removed from the chemical library.

### Hi-speed VS by applying Hypo1_FRED_SAHA-3

#### Access to the VS pipeline and its usage

The pharmacophore model (i.e. Hypo1), the ligand-induced-fit HDAC3 model (i.e. SAHA-3), and the manual related to the thoroughly validated VS pipeline were accessed and downloaded by following the link, i.e. https://www.dropbox.com/sh/5w9d6y8m77hk9pb/AAA2QoItV7wEeFS4p1ZyQzMda?dl=0. The library of drug-like chemical entities was the input of the VS pipeline. They were firstly filtered by Hypo1, and the remaining ones were then submitted for molecular docking against SAHA-3 as well as scoring by Chemgauss4.

#### Hypo1-based pharmacophore filtering

Filtering by the pharmacophore model, i.e. Hypo1 was the first step of our pipeline. Prior to the pharmacophore filtering, a maximum of 100 conformations were generated for each drug-like chemical entity by the “FAST” algorithm implemented in “Build 3D Database” module of DS 2016, which led to the construction of a multi-conformer compound database. During the pharmacophore filtering, all the conformers in the database were matched to Hypo1 by a rigid fit algorithm, i.e. “FAST” algorithm in the “Search 3D Database” module of DS 2016. A FitValue score of each conformer was calculated according to their matching degree to Hypo1 and the conformer of the highest FitValue score was retained for each chemical entity. All the chemical entities that fit Hypo1 were submitted to the second step of the VS pipeline, i.e. molecular docking-based filtering.

#### FRED docking against SAHA-3

SAHA-3 was the optimal receptor model for enriching HDAC3Is. Every chemical entity that passed the pharmacophore filter was firstly converted to multiple three-dimension conformations by OMEGA (version 2.5.1.4; OpenEye Scientific Software, Inc. , Santa Fe, NM, USA)^43^. In pose generation, the maximum number of conformations for each chemical entity was set to the default value of 200. Then, the pool of multiple conformations was docked against the binding site of SAHA-3 by FRED program (now OEDocking, version 3.0.1; OpenEye Scientific Software, Inc., Santa Fe, NM, USA)^44–46^. All the binding poses of each chemical entity were scored by the inherent scoring function of FRED, i.e. Chemgauss4, while only the top-scoring pose was outputted. The chemical entities submitted for molecular docking were ranked according to the FRED Chemgauss4 scores. The 10% top-ranking chemical entities were defined as potential hits.

### Construction of a knowledge-based PF by in-house QSAR modelling approach to facilitate compound cherry-picking

#### Generation of a binding pose set for building PF

OMEGA generated a default 200 conformers for SAHA based its chemical structure. Then FRED docked SAHA from the multi-conformer pool against the binding site of the HDAC3 receptor model, i.e. SAHA-3. From all the binding poses, 1000 top-scoring ones were retained to constitute a binding pose set.

#### Quantitative description of binding poses: classes and features

As a dependent variable for binary QSAR modelling, the class of each pose in the pose set must be pre-defined, i.e. 1 for native-like poses and 0 for pose decoys. The heavy-atom root mean square deviation (RMSD), which measured the difference in terms of atom coordinates between each binding pose and the “native” pose from the HDAC3/SAHA complex model, was used to determine native-like poses and pose decoys. If the RMSD value of a pose was no greater than 4 Å, then it was native-like, otherwise it was a pose decoy. According to the criteria, a protocol that contained a built-in “RMSD calculator” component as well as a customized “class determination” component was constructed by Pipeline Pilot (version 7.5; Accelrys Software, Inc., San Diego, CA, USA). With this protocol, 1000 poses were grouped into 603 native-like poses and 397 pose decoys.

In addition to pose classes, the features of binding poses must be described quantitatively as well. Since differences of those binding poses lay in the protein-ligand interactions instead of the chemical structure itself, the unique descriptors able to characterize the protein–ligand interfaces, i.e. PL/MCT-tess descriptors were applied here. According to the predefined rule, the collection of PL/MCT-tess descriptors included 554 frequently observed types, corresponding to 554 independent variables for QSAR modelling. The calculation of PL/MCT-tess descriptors for a given protein-ligand interface had been implemented in the ENTESS program (https://github.com/moggces, accessed in Oct. 2016) and the algorithms are described as follows. At first, the protein–ligand (PL) interface was partitioned into multiple Delaunay tetrahedrons with protein and ligand atoms as vertices^47^. Then, protein–ligand pairwise atomic potentials based on maximal charge transfer (MCT) was calculated for each Delaunay tetrahedron^48^ ^,^ ^49^, in which the distance between the protein atom and the ligand atom was considered. Subsequently, the sum of the pairwise atomic potentials from multiple Delaunay tetrahedrons of the same type was computed and assigned to the corresponding PL/MCT-tess descriptor type. For those types that did not exist in the specific protein-ligand interface, zero was assigned. For more details about the calculation of PL/MCT-tess descriptors, readers are recommended to refer to our earlier publication by Hsieh et al.^40^.

By the above calculations, the 1000 binding poses from molecular redocking were converted to a 1000 by 555 matrix, where 1000 represented 1000 records while 555 referred to 554 PL/MCT-tess descriptor types plus 1 pose class.

#### Binary QSAR modelling to construct PF

That the ratio of native-like poses to pose decoys was less than 2 (i.e. 603/397) indicated the pose distribution in the dataset was balanced. Therefore, no down-sampling strategy was applied prior to data partition for this case. The pose set was randomly divided into a training set (80% poses) for model building and a test set (20% poses) for model validation. As a result, the training set consisted of 318 pose decoys and 482 native-like poses, while the test set was made up of 79 pose decoys and 121 native-like poses. For binary QSAR modelling, the library for support vector machines (LibSVM, http://www.csie.ntu.edu.tw/∼cjlin/libsvm/, accessed in Jun. 2016.) was used^50^. Based on the training set, grid search for the optimal parameters of radial basis function, i.e. C and γ was performed by five-fold cross-validation (CV). Trained with the best pair of parameters, i.e. (2, 0.0078125) for (C, γ), the model achieved a CV accuracy of 86.75% (cf. Figure S1). As a means of validation, this model was used to predict the class of each pose in the test set and its prediction accuracy was 87%. Due to its high CV accuracy and prediction accuracy, this binary QSAR model, i.e. PF was deemed feasible for pose classification.

### Compound cherry-picking aided by the PF

#### Construction of a multi-pose database of potential hits

Two docking programs, i.e. FRED and AutoDock Vina, were used to generate multiple binding poses by docking the potential hits against the HDAC3 receptor model, respectively. As for FRED, A maximum number of 30 binding poses were kept for each potential hit according to the binding affinity predicted by the Chemgauss4 score. The other parameters were set the same as those in the fast docking step of the VS pipeline. As for Vina, the binding site was defined as a cuboid of 28 Å × 28 Å × 32 Å in size and centred on the cognate ligand of the complex model. The maximum number of binding poses for each potential hit was set as 100, and the maximum energy difference between the best binding mode and the worst displayed was 9 kcal/mol. The free energy of each binding pose was estimated by the inherent scoring function of Vina.

#### Automated inspection of binding modes and scoring of “native” binding pose

Firstly, PF was used to automatically inspect the binding modes and select native-like poses for each potential hit. To be specific, all the binding poses along with its counterpart residues were characterized by PL/MCT-tess descriptors. Based on the descriptors, the binding poses were classified into native-like or decoy poses (or irrelevant poses) by the constructed PF. Secondly, the native-like poses of each potential hit were ranked according to the corresponding scores (or estimated free energies) and the top-scoring binding pose was considered as the “native” pose of that potential hit. The above steps were applied to the analysis of binding poses generated by FRED and Vina, respectively.

#### Compound ranking and clustering

The potential hits were sorted by the Chemgauss4 scores and Vina Scores of their “native” poses, respectively, which generated two rank-ordered lists. The top 202 potential hits from both lists were kept and then merged by the Specs ID. By analysing the chemical structures of 65 overlapping compounds, 12 Specs compounds were cherry-picked. Among them, 11 commercially available compounds were purchased from Compound Handling B.V. (Zoetermeer, South Holland, The Netherlands) and submitted for bioassay.

### *In vitro* HDAC inhibition assay

#### Reagents and pretreatment

All the full-length recombinant human GST-tagged HDACs, i.e. HDAC3/NCoR2 (residues 237-489), HDAC1, HDAC2, HDAC8, HDAC4 and HDAC6) were purchased from SignalChem (British Columbia, Canada). The substrates for HDAC3/NCoR2, HDAC1, HDAC2 were 10 μM Ac-Leu-GlyLys(Ac)-AMC, while the substrates for HDAC4 and HDAC6 were 2 μM Ac-Leu-Gly-Lys(Tfa)-AMC and 2 μM Ac-Leu-GlyLys(Ac)-AMC, respectively. For HDAC8, the substrate was 2 μM Ac-Leu-Gly-Lys(Tfa)-AMC. All the HDACs substrates were synthesized by GL Biochem (Shanghai, China). Trypsin was purchased from Sangon Biotech (Shanghai, China). The assay buffer was composed of 25 mM Tris, pH 8.0, 1 mM MgCl_2_, 0.1 mg/ml BSA, 137 mM NaCl and 2.7 mM KCl. The purchased compounds were initially dissolved in the dimethyl sulfoxide (DMSO) to make DMSO stock solutions (10 mM), and then diluted to ten times the tested concentration(s) by HDAC assay buffer.

#### Bioassay protocol

Firstly, the compounds were assayed for their HDAC3 inhibitory activity. For preliminary assay, the tested concentrations were 10 μM. For secondary assays, the activity of each compound was tested at nine concentrations from 10 nM to 100 μM. Only the identified HDAC3 inhibitors were further tested for their activity against a panel of other HDACs. The protocol for HDAC bioassay is as follows^51^. The bioassay was performed in blank low-binding Nunc 96-well microtiter plates. In each well, 25 μL HDAC assay buffer (including fluorogenic substrate), 10 μL compound solutions and 15 μL HDAC were added and the plate was pre-incubated at 37 °C for 30 min. After that, the HDAC3 assay developer, i.e. trypsin (50 μL, 0.4 mg/mL) was added to each well, and the plate was incubated for another 30 min at the temperature of 37 °C. The fluorescence was then measured at an excitation of 360 nm and an emission wavelength of 460 nm by a Perkin-Elmer Enspire plate reader. In the bioassay, the assay with SAHA or TSA (for HDAC4) in place of the tested compound was used as a positive control, while the assay without any compound was a negative control. The activity (%) was calculated to measure the inhibitory effect of the compound according to the following formula, i.e. activity% = {(FL_cmpd_ – FL_blank_)/(FL_no_cmpd_ – FL_blank_)} × 100%. After the secondary bioassay, the IC_50_ value was calculated using nonlinear regression with normalized dose-response fit in GraphPad Prism 5 software (GraphPad Software Inc., La Jolla, CA, USA). All the assays were performed in triplicate (for HDAC3) or duplicate (for other HDAC isoforms).

### Substructure search

The protocol in Pipeline Pilot that implemented substructure search was used for our purpose. The core scaffold named N-(2-hydroxyphenyl)benzamide of the hit compound was defined as the substructure. In substructure search, compounds with any substituted N-2-hydroxyphenyl group (e.g. N-(2-methoxyphenyl) group or N-(akyl-2-hydroxyphenyl) group) were not considered. Besides, only the structures in which the phenyl group of benzamide was substituted with a fragment similar to the phthalamide group in size were submitted for bioassay.

## Results and discussions

### The workflow and outcome of hit identification

**Figure 2.**
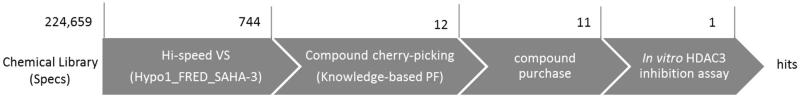
The workflow for the discovery of novel HDAC3 inhibitors. The number refers to the amount of chemical entities that entered the next component of the workflow.

The outline for the discovery of novel HDAC3Is in this study is described in Figure 2. The initial number of compounds in the Specs chemical library used for VS was 212,531. The preparation of compounds and Lipinski’s “rule of 5” filtering rendered a total of 224,659 chemical entities (i.e. both protonated forms and stereoisomers). The first step of our previously constructed pipeline, i.e. Hypo1-based pharmacophore filtering, identified 7484 chemical entities (5768 unique Specs compounds) that matched Hypo1, with FitValue scores ranging from 0.00125 to 3.56231. Among them, 40 chemical entities failed in pose generation for their unspecified stereochemistry while four chemical entities were not able to fit in the binding site due to their molecular sizes. Therefore, only 7440 chemical entities were successfully “positioned” in the binding site of the HDAC3 structural model. We picked 10% of them, i.e. 744 chemical entities (664 unique compounds) as potential hits. For these chemical entities, the minimum of FRED Chemgauss4 scores was −14.8607, while the maximum was −9.83381.

**Figure 3.**
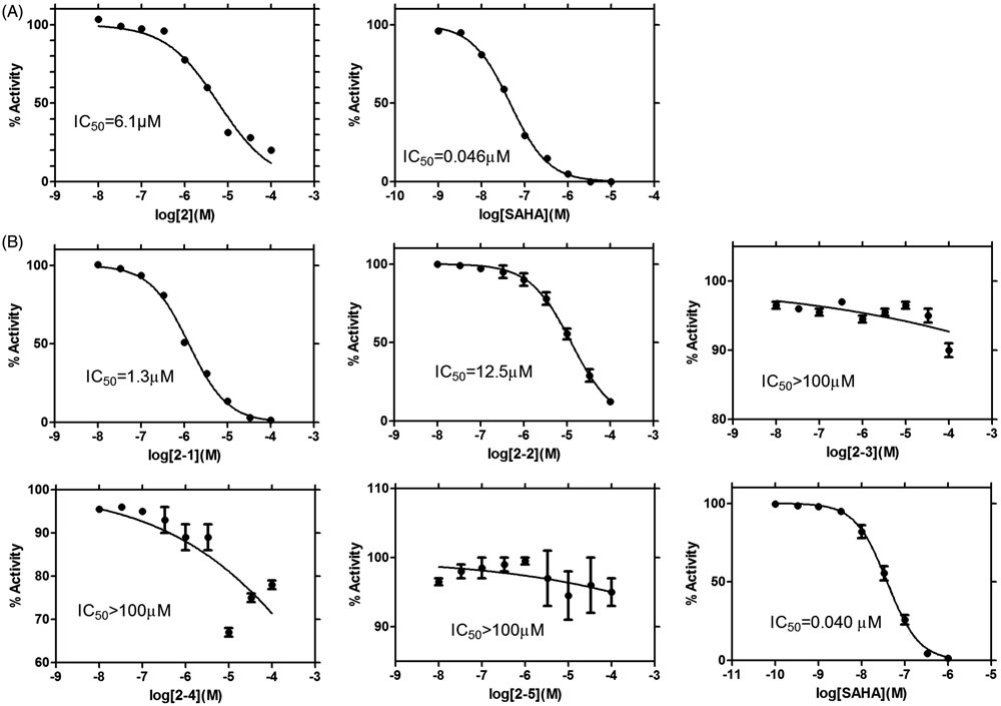
Dose response curves of **2** (A) and its derivatives from substructure search (i.e. **2–1** ∼ **2–5**, B). The dose response curves of the positive drug (i.e. SAHA) in two rounds of bioassays are also shown.

To facilitate cherry-picking of compounds from the set of 744 potential hits, we constructed a knowledge-based PF specific to HDAC3 by using our unique cheminformatics method^40^. The PF was trained based on our model of HDAC3 bound to SAHA^39^, and it was able to classify poses in a CV accuracy of 86.75% and a prediction accuracy of 87%. We applied the PF by coupling it with docking program(s). To boost confidence, we used more than one docking programs for our purpose, i.e. FRED and Vina. Initially, FRED generated a set of 22,320 binding poses while Vina outputted a set of 14,873 poses for all the potential hits. Then, PF automatically recognized the “native-like” binding poses in each set by using the values of PL/MCT-tess descriptors as input. Subsequently, the inherent scoring function of each docking program identified “native” poses by taking the predicted binding free energies into account. We ranked the potential hits by the Chemgauss4 scores and Vina scores of their “native” poses, respectively and selected 202 top potential hits from each list. We further analysed 66 overlapping potential hits (65 unique Specs compounds) by inspecting the chemical structures. As 41 compounds shared the fragment of 1H-1,2,4-triazol-3-amine or 2H-tetrazol-5-amine, we merely selected 3 representative compounds from them, i.e. AN-465/43370023, AM-900/15050012 and AN-465/43369338 (cf. Table S1). By clustering the remaining 24 compounds into five clusters, we picked no more than three compounds from each cluster, which resulted in another set of nine diverse compounds. Since one compound (Specs ID: AN-652/43024757) was not commercially available at that time, 11 compounds were purchased and tested for their inhibitory effect on HDAC3 *in vitro*. Among them, compound **2** showed 63.5 ± 1.5% inhibition of HDAC3 in the preliminary assay (cf. Table 1 and Figure S2). A follow-up bioassay determined its IC_50_ value as 6.1 μM (cf. Figure 3(A) and Table 2). In order to identify more hits of the same chemotype, we further searched for the derivatives of compound **2** from the Specs library by substructure search. Five compounds that met the criteria for substructure search were retrieved and purchased. Among them, two compounds, i.e. **2–1** and **2–2** showed HDAC3 inhibition, with IC_50_ values of 1.3 and 12.5 μM, respectively (cf. Figure 3(B) and Table 2).

**Table 1.** Eleven cherry-picked and purchased compounds, and their inhibitory activity on HDAC3 in terms of inhibition rate (%) at 10 μM.

ID	Specs ID	Chemical structure	% inhibition rate (mean ± SD)^a^
1	AK-968/40357504		1.1 ± 1.8
2	AN-979/41971160		63.5 ± 1.5
3	AN-989/41695943		8.4 ± 2.4
4	AO-022/43453016		3.7 ± 1.8
5	AK-968/41024638		17.7 ± 0.7
6	AO-081/41888698		0.8 ± 0.9
7	AN-652/13748008		1.0 ± 1.4
8	AN-023/15593034		9.0 ± 2.7
9	AN-465/43370023		0.5 ± 1.5
10	AM-900/15050012		4.1 ± 2.5
11	AN-465/43369338		8.3 ± 1.3
Positive control	SAHA		99.1 ± 1.8

a Percentage inhibition was tested under the concentration of 10 μM, and the values were the average of triplicates.

**Table 2.** Inhibitory effects of the hit compound **2** and its derivatives from substructure search on HDAC3.

Tested compounds				Nearest neighbour		
Name	SPECS ID	Chemical structure	IC_50_ (μM)	CHEMBLID	Chemical structure	*Tc* value
**2**	AN-979/41971160		6.1	CHEMBL405072		0.317
**2–1**	AQ-390/42122119		1.3	CHEMBL235191		0.297
**2–2**	AN-329/43450111		12.5	CHEMBL236061		0.355
**2–3**	AN-329/43449880		>100	–		
**2–4**	AK-918/12439013		>100	–		
**2–5**	AK-918/11144035		>100	–		

The nearest neighbour of each hit compound from known HDAC3Is (IC_50_ < 15 μM) in ChEMBL23 is listed.

To explore the structural novelty of the three hit compounds, we identified their respective nearest neighbours by (1) collecting a pool of reported HDAC3Is whose IC_50_ values were no greater than 15 μM from ChEMBL23 (https://www.ebi.ac.uk/chembl/, accessed in Jun. 2017)^52^, (2) calculating Tanimoto coefficient (*Tc*) similarity based on FCFP_6 fingerprints of all active compounds to each hit compound. As shown in Table 2, the nearest neighbour of compound **2** was CHEMBL405072, whose *Tc* value equalled to 0.317. For compounds **2–1** and **2–2**, their nearest neighbours were CHEMBL235191 (*Tc* = 0.297) and CHEMBL236061 (*Tc* = 0.355), respectively. Notably, the most unique structural features of the hit compounds were the substituent fragments in the phenyl group of benzamide. The replacement of the primary amine group by a hydroxyl group is the other noteworthy feature. The low *Tc* values and unique features of the three hit compounds indicated of chemical diversity compared with the reported HDAC3Is.

### The role of Hypo1_FRED_SAHA-3 and the knowledge-based PF in the hit identification

The identification of novel hit compounds validated the efficacy of our workflow for HDAC3Is discovery. Since the workflow was composed of two main components, i.e. Hypo1_FRED_SAHA-3 pipeline and the knowledge-based PF, it became necessary to analyse the individual performance of each computational tool.

As for the Hypo1_FRED_SAHA-3 pipeline, our hit compound **2** was right in the set of 744 potential hits, indicating that that pipeline was able to effectively place active compound(s) at the top of the screen list from a large-scale chemical library, e.g. the Specs chemical library composed of 212,531 compounds. Since the purchase and bioassay of 744 compounds still cost too much, we constructed the knowledge-based PF and coupled it with FRED/Chemgauss4 and Vina/Vinascore for the cherry-picking of a small-size compound set.

As for PF, it was designed to provide an automatic and fast way to inspect binding modes/poses and avoid subjective decision as greatly as possible. When coupled with FRED, it excluded 748 irrelevant poses from the pose set generated by FRED, 11 of which were also poses of lowest Chemgauss4 scores, i.e. top-scoring poses. Due to the exclusion of the top-scoring poses and the use of the score of the “native” pose instead of the top score for the compound ranking, the rank orders of 744 potential hits were different from those prior to the use of the PF. Table S2 lists the 11 potential hits whose native poses were not the top-scoring poses. Clearly, the ranks of the 11 potential hits decreased due to the use of PF. Since we did not select these potential hits (ranked behind 202) for further analysis, the PF coupled with FRED did not affect our final decision in the compound cherry-picking. Unlike coupling with FRED/Chemgauss4, the PF affected the outcome of Vina significantly. It excluded 3,430 irrelevant poses from the set of 14,873 binding poses generated by Vina, of which 144 poses corresponded to top-scoring poses of 144 potential hits. Table S3 lists the details of 144 potential hits, i.e. the name of each potential hit as well as its respective score and rank order when sorted by the top-scoring pose and the native pose. In our workflow, we selected 202 potential hits from the compound list based on the VinaScore of the native pose. The cutoff in terms of VinaScore to select 202 potential hits was −8.1. Prior to the filtering of irrelevant poses, 209 potential hits represented by the top-scoring pose were scored less than or equal to −8.1. Therefore, the number of potential hits dropped from 209 to 202, which indicated seven potential hits were not included in the compound list of Vina due to the use of PF (cf. Table S3). As it affected the list of overlapping compounds between FRED and Vina, the PF played a role in decision made for compound cherry-picking.

### The hit compounds and preliminary SAR

**Figure 4.**
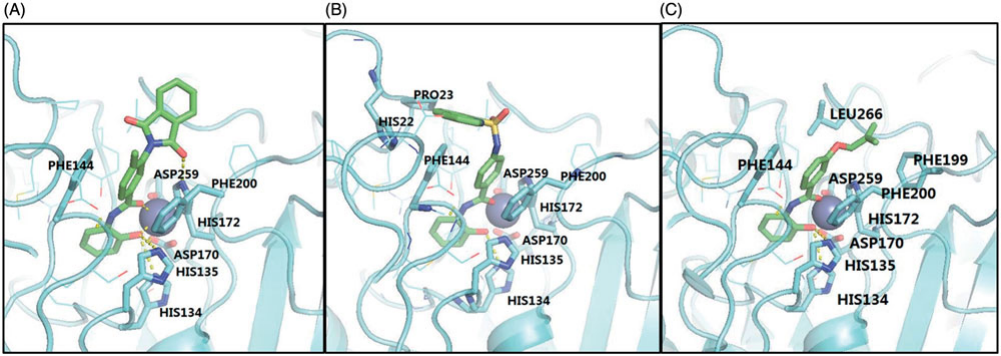
The modes of compound **2** (A), **2–1** (B) and **2–2** (C) bound to HDAC3, predicted by FRED. Colour codes: light blue, HDAC3; green, hit compounds; blue sphere, zinc ion.

In the first-round screening, compound **2** inhibited HDAC3 at the concentration of 10 μM. Though the low FitValue score of this compound (0.74316, cf. Table S1) indicated it partially matched the pharmacophore model, i.e. Hypo1 of the VS pipeline, it bound to our receptor model, i.e. SAHA-3 in a favorable way. Figure 4(A) clearly shows the unique N-(2-hydroxyphenyl)benzamide group is involved in the major interactions between compound **2** and HDAC3. To be specific, (1) the 2-hydoxyl group of benzene and the carbonyl group of benzamide on the ligand side, together with Asp259 and Asp170 on the protein side potentially formed coordination bond with the zinc ion; (2) the benzene of the benzamide was sandwiched between Phe144 and Phe200 by forming π-π stacking. As mentioned before, we used the N-(2-hydroxyphenyl)benzamide group as a core scaffold for substructure search. The pivotal role of this chemical group in protein-ligand interaction that we pointed out here provided a strong reason for that decision.

All the five derivatives of compound **2** contained the N-(2-hydroxyphenyl)benzamide core, however, only compounds **2**, **2–1** and **2–2** were active for HDAC3 (cf. Table 2). This result implied the capping groups (e.g. the phthalamide group) had significant effects on the bioactivity of compounds. For instance, (1) the phthalamide group of compound **2** formed a hydrogen bond with Asp59, which stabilized the ligand binding. (2) Though all the capping groups of **2–1**, **2–4** and **2–5** were predicted to be located in a side pocket surrounded by Pro23, His22 and Phe144, the quinoline group of **2–1** may form π–π interaction while the others can only form weaker σ–π conjugation effect (cf. Figures 4B and S3B/C). The weak σ–π conjugation effect may not able to ensure strong ligand binding thus caused the loss of bioactivity. (3) Due to the substitution of 2-methylallyl-oxyl group at the ortho-position, the core scaffold of compound **2–3**, i.e. the N-(2-hydroxyphenyl)benzamide failed to stretch into the catalytic site (cf. Figure S3A). As a result, it showed no inhibitory effect while the meta-substituent (i.e. compound **2–2**, cf. Figure 4C) was active against HDAC3.

Based on the current data, we were able to get insight into the preliminary SAR for this class of HDAC3Is. The N-(2-hydroxyphenyl)benzamide group represented the major structural feature for this class of HDAC3Is, but this group did not ensure biological activity. The introduction of groups of conjugation effect (e.g. aromatic rings) to the capping group may be beneficial for activity.

### Selectivity profile of the hit compounds

Isoform selectivity as an indicator of safety is essential to the application of HDAC3Is for the treatment of diseases. Therefore, we tested our three hit compounds against a panel of important HDACs targets, including HDAC1, HDAC2 and HDAC8 within Class I HDACs, HDAC4 in Class IIA HDACs as well as HDAC6 within Class IIB HDACs. As listed in Table 3, all the three hit compounds showed potent inhibition against HDAC1, HDAC2 and HDAC3, while they were not active against HDAC4, HDAC6 and HDAC8 at the concentration of 100 μM (cf. Figure S4). These data indicated the identified hit compounds were sub-class (or HDAC1/2/3) selective inhibitors. Among them, **2–1** was the most potent as its IC_50_ values were approximately 1 μM against HDAC1, HDAC2 and HDAC3. **2–2** was less potent than **2–1**, as the IC_50_ value for HDAC3 was 12.5 μM while the IC_50_ values for HDAC1 and HDAC2 were 16.6 μM and 29.3 μM. To be noted, the potency of the compound **2** for HDAC3 (IC_50_ = 6.1 μM) was 2.1-fold that for HDAC1 (IC_50_ = 12.7 μM) and 4.1-fold that for HDAC2 (IC_50_ = 24.8 μM), which indicated the compound 2 showed the best selectivity profile among all hit compounds (cf. Table 4).

**Table 3.** Activity profiling of three hit compounds, i.e. **2**, **2–1** and **2–2** against a panel of HDAC isoforms.

Name	IC_50_ (μM)					
	HDAC3	HDAC1	HDAC2	HDAC8	HDAC4	HDAC6
**2**	6.1	12.7	24.8	>100	>100	>100
**2–1**	1.3	0.957	1.78	>100	>100	>100
**2–2**	12.5	16.6	29.3	>100	>100	>100
Positive drug	0.043^a^	0.0633	0.173	4.33	1.37^b^	0.0222

^a^T he average from two independent tests.

b TSA instead of SAHA was used as a positive drug for HDAC4.

**Table 4.** Selectivity index (SI) of three HDAC3 inhibitors over other HDAC isoforms.

Name	Selectivity index (SI)^a^				
	HDAC1/HDAC3	HDAC2/HDAC3	HDAC8/HDAC3	HDAC4/HDAC3	HDAC6/HDAC3
**2**	2.1	4.1	>16	>16	>16
**2–1**	0.74	1.4	>77	>77	>77
**2–2**	1.3	2.3	>8	>8	>8

a SI equals to the quotient of IC_50_ value for one HDAC isoform to that for HDAC3.

## Conclusions

In the present study, we made attempts to validate our previously designed VS pipeline by applying it to screening Specs chemical library for HDAC3Is. In practice, we realized that it still remained a tough decision making to select a small number of compounds from 744 potential hits that passed through the VS pipeline. To this end, we developed the knowledge-based PF and coupled it with FRED and Vina, which functioned to automatically and rapidly inspect the binding modes and filtered the irrelevant binding poses. The new workflow mainly composed of the VS pipeline and the novel knowledge-based PF rendered 11 commercially available potential hits. By compound purchase and *in vitro* HDAC3 inhibition bioassay, we have discovered one of them, i.e. compound **2** (AN-979/41971160) was able to inhibit HDAC3 (IC_50_ = 6.1 μM). The performance analysis of the VS pipeline and the PF indicated the former was indeed able to enrich active compounds at the top of the compound list while the latter facilitated in cherry-picking of compounds by re-ranking compounds. In summary, the current work has experimentally validated the efficacy of our prior VS pipeline. Moreover, it provided the scientific community with a more competent workflow that included the automated inspection of binding modes.

As a medicinal chemistry effort toward HDAC3Is discovery, we further performed substructure search using the N-(2-hydroxyphenyl)benzamide group. Among five commercially available derivatives of compound **2**, **2–1** (AQ-390/42122119) and **2–2** (AN-329/43450111) showed inhibitory effects on HDAC3. The scaffold analysis indicated this class of HDAC3Is was diverse compared to reported ones. In addition, the preliminary SAR analysis based on molecular docking has uncovered that the introduction of groups of conjugation effect (e.g. aromatic rings) as the capping group was essential to maintain the activity of this class of HDAC3Is. We also studied the *in vitro* activity of the three hit compounds for HDAC1/2/8 (class I), HDAC4 (class IIA) and HDAC6 (class IIB). Interestingly, all the three hit compounds were HDAC1/2/3 selective, of which compound **2** showed the best selectivity profile.

The present study is our continuous effort on the development and application of new cheminformatics methods for the discovery of HDAC3Is. It not only validated our earlier VS pipeline experimentally, but also optimized the workflow by providing an alternative way to visual inspection. Due to the broad applications of HDAC3Is, the newly identified hit compounds will be interesting for synthetic medicinal chemists to follow up. Next work will focus on the improvement of potency by structural optimization of the hit compounds according to the gained preliminary SAR.

## Supplementary Material

IENZ_1437156_Supplementary_Material.pdf
