# Supplementary material for: The discovery of novel HDAC3 inhibitors via virtual screening and in vitro bioassay
Source: J Enzyme Inhib Med Chem. 2018 Feb 21;33(1):525–35. doi: 10.1080/14756366.2018.1437156 (PMC5978667; doi:10.1080/14756366.2018.1437156)
Supplement: IENZ_1437156_Supplementary_Material.pdf [file IENZ_A_1437156_SM3086.pdf]

SUPPORTING INFORMATION

The discovery of novel HDAC3 inhibitors via virtual screening and *in vitro* bioassay

Jie Xia*[1], Huabin Hu[1], Wenjie Xue[1], Xiang Simon Wang[2] and Song Wu *[2]

[1] *State Key Laboratory of Bioactive Substance and Function of Natural Medicines, Department of New Drug Research and Development, Institute of Materia Medica, Chinese Academy of Medical Sciences and Peking Union Medical College, Beijing 100050, China.;* [2] *Molecular Modeling and Drug Discovery Core Laboratory for District of Columbia Center for AIDS Research (DC CFAR), Department of Pharmaceutical Sciences, College of Pharmacy, Howard University, Washington, DC 20059, U.S.A.*

*Correspondence should be addressed to J.X. (jie.william.xia@hotmail.com) and S.W. (ws@imm.ac.cn)

Table S1. The chemical structures, FitValue scores, PF_Chemgauss4 scores (Chemgauss4 scores after PF filtering), PF_Vina scores (Vina scores after PF filtering) as well as commercial availability of compounds that were cherry-picked from the Specs library. The bold entry refers to the hit compound.

ID	Specs ID	chemical structure	FitValue	PF_Chemgauss4	PF_Vina (kcal/mol)	Commercially available (Y/N)
1	AK-968/40357504	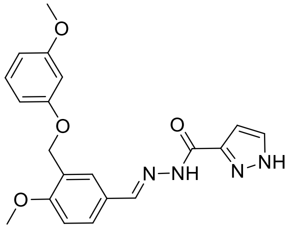	0.682217	-13.7209	-9.2	Y
**2**	**AN-979/41971160**	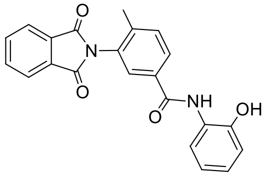	**0.74316**	**-12.5627**	**-8.1**	**Y**
3	AN-989/41695943	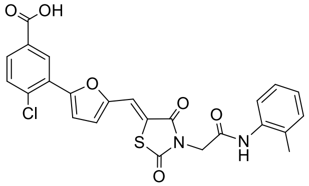	0.45563	-12.671	-8.5	Y

4	AO-022/43453016	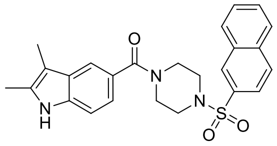	1.72774	-11.9285	-9.2	Y
5	AK-968/41024638	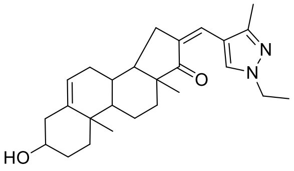	2.83669	-11.7728	-8.1	Y
6	AO-081/41888698	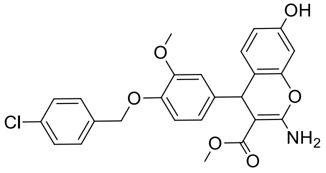	1.53288	-11.4509	-8.3	Y
7	AN-652/13748008	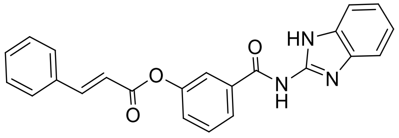	0.962872	-12.0009	-8.4	Y
8	AN-023/15593034	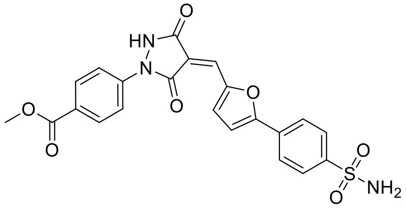	0.820441	-13.1009	-8.7	Y

9	AN-465/43370023	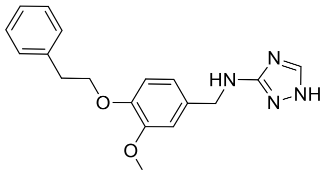	1.14038	-12.663	-8.6	Y
10	AM-900/15050012	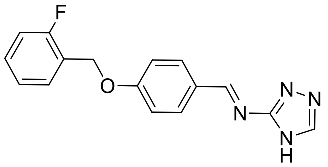	0.823326	-14.8607	-9.9	Y
11	AN-465/43369338	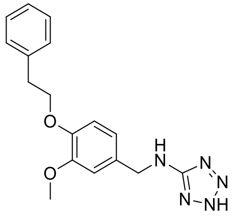	1.37083	-12.2554	-8.7	Y
12	AN-652/43024757	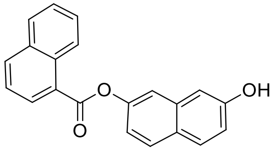	0.890428	-11.4295	-8.9	N

Table S2. 11 chemical entities whose top-scoring poses from FRED/Chemgauss4 were not predicted as "native-like". The scores and ranks of their top-scoring poses and native poses are listed.

No.	ID of chemical entity	Top-scoring Pose		"native" pose		
		score	rank[a]	Index[b]	score	rank
1	AH-487/40686896-63145	-10.9286	203	2	-10.758	251
2	AH-487/40686750-63102	-10.8851	214	3	-9.67662	742
3	AN-465/42246653-136152	-10.8385	228	2	-10.8256	228
4	AH-487/41731030-64479	-10.773	252	3	-10.735	262
5	AN-465/41587333-135052	-10.7689	253	2	-10.2444	464
6	AQ-149/41812870-204818	-10.6095	312	2	-9.58038	743
7	AK-968/41923320-110364	-10.2398	470	2	-10.1556	524
8	AQ-149/41812535-204776	-10.2154	489	2	-9.77257	741
9	AN-329/43211399-129002	-10.1626	524	2	-9.94469	658
10	AS-871/43478038-217416	-10.0938	566	3	-9.98415	629
11	AK-968/40282836-102790	-9.84685	736	4	-9.20708	744

[a] The maximal rank is 744.

[b] The maximal index is 30.

Table S3. 144 chemical entities whose top-scoring poses from Vina/VinaScore were not predicted as "native-like". The scores and ranks of their top-scoring poses and native poses are listed. 7 potential hits that dropped out of the compound list of Vina were highlighted in bold.

index	Name of potential hits	top-scoring pose		"native" pose		
		VinaScore	rank	Index	VinaScore	rank
1	AN-465/41587333-135052	-9	32	2	-8.6	91
2	AH-487/40686896-63145	-8.9	49	5	-8.1	174
3	AK-968/41923358-110523	-8.6	93	2	-8.5	107
**4**	**AG-690/10008047-40510**	**-8.5**	**109**	**3**	**-7.7**	**286**
5	AK-968/15604952-99866	-8.5	109	2	-8.1	174
6	AK-968/41923540-110810	-8.4	125	2	-8.3	135
7	AQ-390/41639738-207079	-8.4	125	6	-8.2	157
8	AN-465/41587275-135026	-8.3	140	2	-8.3	135
**9**	**AO-081/41888559-166161**	**-8.3**	**140**	**2**	**-8**	**203**
**10**	**AK-968/37129204-101881**	**-8.3**	**140**	**2**	**-7.7**	**286**
**11**	**AO-299/40799417-167063**	**-8.3**	**140**	**2**	**-8**	**203**
**12**	**AK-968/41923320-110341**	**-8.3**	**140**	**3**	**-7.6**	**332**
13	AG-690/36723001-54523	-8.2	165	2	-8.2	157
**14**	**AK-968/41923320-110441**	**-8.2**	**165**	**2**	**-7.5**	**363**
15	AK-968/40709254-105455	-8.2	165	2	-8.2	157
**16**	**AO-080/43441553-163156**	**-8.1**	**182**	**2**	**-7.7**	**286**
17	AJ-292/41945407-72892	-8	210	2	-8	203
18	AN-329/43448279-130940	-7.9	240	2	-7.9	235
19	AK-968/41923320-110364	-7.9	240	2	-7.5	363
20	AO-081/42037276-166395	-7.9	240	4	-7.9	235
21	AG-205/36566045-30218	-7.9	240	2	-7.7	286
22	AO-080/43379180-163057	-7.9	240	2	-7.5	363
23	AH-487/42481569-66244	-7.8	263	2	-7.8	255
24	AP-866/42031451-195226	-7.8	263	8	-7	530
25	AQ-390/43363894-207897	-7.8	263	4	-7.3	429
26	AO-022/43451970-157347	-7.8	263	2	-7.7	286
27	AN-465/43411356-140235	-7.7	297	3	-7.7	286
28	AK-968/41024608-108044	-7.7	297	3	-7.5	363
29	AK-968/40709584-105569	-7.7	297	4	-6.8	601
30	AN-465/43411026-139902	-7.7	297	4	-7.5	363
31	AP-906/42126945-196419	-7.7	297	2	-7.7	286
32	AG-690/40752644-58528	-7.6	341	2	-7.4	400
33	AP-970/41681657-198327	-7.6	341	2	-7.6	332
34	AP-970/41681528-198310	-7.6	341	4	-7.1	504
35	AK-968/41923371-110628	-7.6	341	2	-7.6	332
36	AP-866/42942059-195270	-7.6	341	4	-7.2	476
37	AN-465/42887664-137736	-7.6	341	2	-7.6	332

38	AK-968/41923320-110410	-7.6	341	3	-7.3	429
39	AS-871/42849822-214325	-7.6	341	2	-7.5	363
40	AG-205/33153037-29080	-7.5	376	3	-7.3	429
41	AO-022/42287969-155924	-7.5	376	3	-7.4	400
42	AE-848/34330044-14512	-7.5	376	2	-7.4	400
43	AN-698/42116797-150508	-7.5	376	2	-7.1	504
44	AN-465/42887665-137737	-7.5	376	2	-7.5	363
45	AN-465/43369921-139490	-7.5	376	2	-7.5	363
46	AP-970/41681519-198305	-7.5	376	8	-6.4	700
47	AO-080/41818325-161837	-7.5	376	4	-7.2	476
48	AP-970/41681662-198332	-7.5	376	2	-7.4	400
49	AP-970/41681512-198299	-7.5	376	2	-7.5	363
50	AN-465/42246675-136174	-7.5	376	2	-7.5	363
51	AK-968/36929014-100978	-7.5	376	2	-7.2	476
52	AK-968/41017066-106929	-7.5	376	3	-7.5	363
53	AN-465/40853754-134553	-7.4	415	3	-7.1	504
54	AG-205/13322065-27304	-7.4	415	4	-7	530
55	AG-690/40752635-58526	-7.3	442	8	-7	530
56	AS-871/42846899-214317	-7.3	442	3	-6.7	634
57	AP-970/41681522-198307	-7.3	442	5	-6.4	700
58	AK-968/41017066-106922	-7.3	442	3	-7.2	476
59	AP-970/41681660-198330	-7.3	442	2	-7.3	429
60	AP-906/42126953-196430	-7.3	442	2	-7.3	429
61	AN-329/43341713-129368	-7.3	442	2	-7.1	504
62	AK-968/40641234-104067	-7.3	442	2	-7	530
63	AO-022/43453190-158480	-7.3	442	2	-7.3	429
64	AQ-149/41812523-204766	-7.3	442	2	-7.3	429
65	AS-871/43478035-217415	-7.3	442	3	-7	530
66	AK-777/12968006-75901	-7.2	493	3	-7.2	476
67	AP-970/42837429-199993	-7.2	493	2	-6.9	571
68	AH-487/40935629-63378	-7.2	493	2	-7	530
69	AQ-360/14045175-206344	-7.2	493	2	-6.7	634
70	AE-641/00605037-8772	-7.2	493	2	-7.1	504
71	AQ-149/43372334-205440	-7.1	521	3	-6.8	601
72	AN-465/41988146-135369	-7.1	521	2	-7	530
73	AK-918/41945408-86038	-7.1	521	4	-6.4	700
74	AN-465/43411194-140072	-7.1	521	2	-7.1	504
75	AP-853/43368077-194422	-7.1	521	3	-7	530
76	AQ-149/43372310-205396	-7.1	521	2	-6.7	634
77	AP-970/41681567-198322	-7.1	521	2	-7.1	504
78	AQ-149/41812692-204804	-7.1	521	2	-7	530
79	AP-970/41681534-198313	-7.1	521	3	-7.1	504
80	AG-664/14117585-35859	-7.1	521	2	-7.1	504
81	AE-641/30153024-10589	-7	548	4	-6.5	681

82	AN-465/43479872-141443	-7	548	3	-6.6	665
83	AN-465/41988158-135371	-7	548	3	-6.8	601
84	AN-329/40239012-122878	-7	548	2	-6.9	571
85	AP-853/43260528-194175	-7	548	3	-6.7	634
86	AK-918/11424054-81346	-7	548	2	-6.9	571
87	AP-970/41681510-198298	-7	548	3	-7	530
88	AG-205/10552028-25928	-7	548	3	-6.8	601
89	AQ-149/43372311-205398	-7	548	3	-6.9	571
90	AN-988/14199004-153373	-7	548	3	-7	530
91	AN-465/41988703-135419	-6.9	588	2	-6.9	571
92	AN-465/42885879-137215	-6.9	588	2	-6.9	571
93	AQ-149/41812916-204824	-6.9	588	2	-6.4	700
94	AG-690/40698078-57436	-6.9	588	2	-6.8	601
95	AQ-149/41812747-204808	-6.9	588	2	-6.9	571
96	AH-487/41954166-65180	-6.9	588	3	-6.9	571
97	AO-854/43457687-178827	-6.9	588	5	-6.6	665
98	AN-465/43411362-140241	-6.9	588	2	-6.9	571
99	AN-329/40614264-123712	-6.9	588	3	-6.7	634
100	AP-853/43368072-194418	-6.9	588	2	-6.8	601
101	AP-263/43370995-185762	-6.9	588	2	-6.8	601
102	AN-329/43211399-129001	-6.9	588	3	-6.7	634
103	AO-365/43473937-171540	-6.9	588	2	-6.9	571
104	AN-329/42158845-126609	-6.9	588	2	-6.9	571
105	AK-968/40282836-102790	-6.9	588	2	-6.9	571
106	AN-465/42243847-135837	-6.8	621	5	-6.6	665
107	AN-465/42889398-138128	-6.8	621	2	-6.5	681
108	AG-690/09166034-39601	-6.8	621	2	-6.8	601
109	AN-465/42243987-135858	-6.8	621	3	-6.8	601
110	AN-967/15488318-152854	-6.8	621	3	-6.3	717
111	AG-690/40751394-58399	-6.8	621	5	-6.4	700
112	AN-652/42205479-146601	-6.8	621	2	-6.8	601
113	AK-968/40369332-103170	-6.8	621	3	-6.6	665
114	AN-329/40239135-122894	-6.8	621	2	-6.6	665
115	AN-329/43449894-132775	-6.8	621	6	-6.4	700
116	AN-329/43211338-128890	-6.8	621	2	-6.7	634
117	AG-690/33362059-52127	-6.8	621	2	-6.8	601
118	AG-664/14117594-35864	-6.8	621	3	-6.5	681
119	AK-968/15254138-96999	-6.7	656	2	-6.5	681
120	AN-465/42243846-135835	-6.7	656	4	-6.5	681
121	AP-853/43416267-194944	-6.7	656	4	-6.3	717
122	AT-057/41488454-218641	-6.7	656	2	-6.7	634
123	AQ-149/41812922-204826	-6.7	656	2	-6.7	634
124	AG-219/09033021-33778	-6.7	656	3	-6.5	681
125	AK-918/41675498-85771	-6.6	684	3	-6.4	700

126	AO-854/43466640-179591	-6.6	684	2	-6.5	681
127	AN-465/43479872-141444	-6.5	697	3	-6.3	717
128	AP-853/43386911-194498	-6.5	697	2	-6.3	717
129	AO-548/42024306-176359	-6.5	697	2	-6.5	681
130	AP-853/43416462-195066	-6.5	697	4	-6.2	730
131	AQ-750/42051590-209115	-6.5	697	2	-6.3	717
132	AN-465/41519712-134905	-6.4	713	2	-6.4	700
133	AK-918/12155017-82628	-6.4	713	2	-6.4	700
134	AP-853/43368071-194417	-6.4	713	2	-6.4	700
135	AP-853/43387181-194539	-6.4	713	2	-6.4	700
136	AQ-149/43372329-205430	-6.4	713	2	-6.4	700
137	AN-465/42887814-137772	-6.4	713	2	-6.3	717
138	AF-399/15539034-18313	-6.3	724	2	-6.3	717
139	AP-853/43261177-194206	-6.2	731	2	-6.2	730
140	AP-853/43368076-194421	-6.2	731	2	-6.2	730
141	AE-848/34512011-14579	-6.2	731	2	-6.2	730
142	AT-057/43231961-219171	-6.1	738	3	-6.1	738
143	AP-853/40871398-193268	-5.7	742	3	-5.6	743
144	AJ-292/40871988-71586	-5.5	744	4	-5.2	744

[a] The maximal rank is 744.

[b] The maximal index is 30.

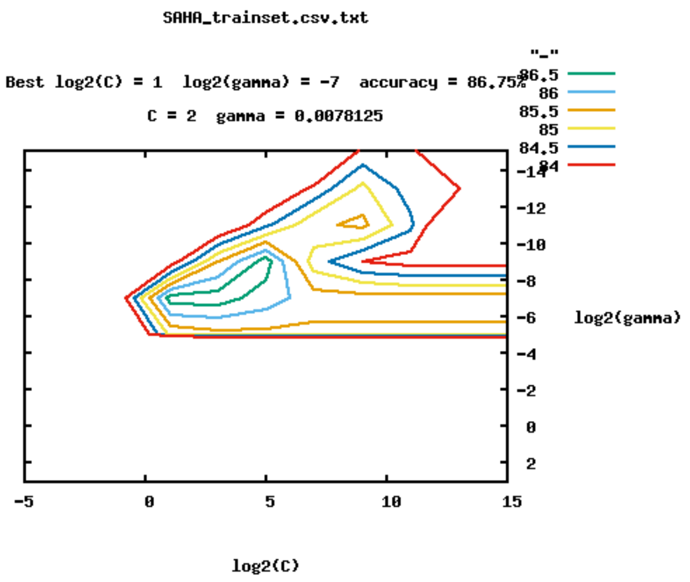

Figure S1. Grid search to find the optimal pair of parameters (C, γ) for model building.

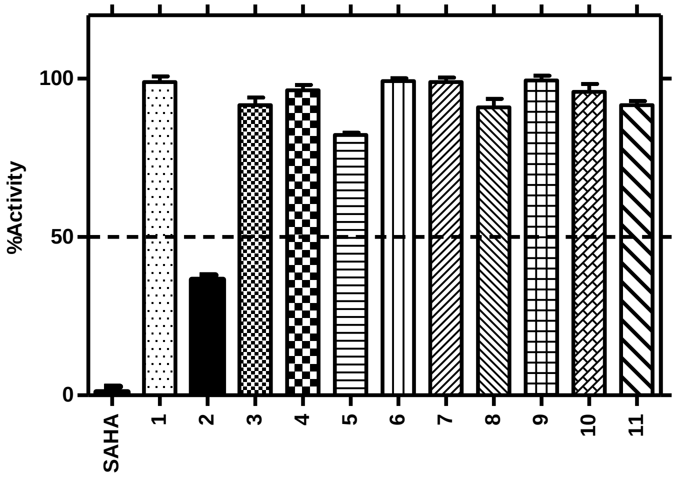

Figure S2. The first-round screening of 11 purchased compounds for HDAC3 inhibitor(s). A compound is regarded as active if the enzymatic activity (%) after the treatment of that compound is less than 50%. The columns filled in black refer to the hit compound. SAHA was used as a positive control.

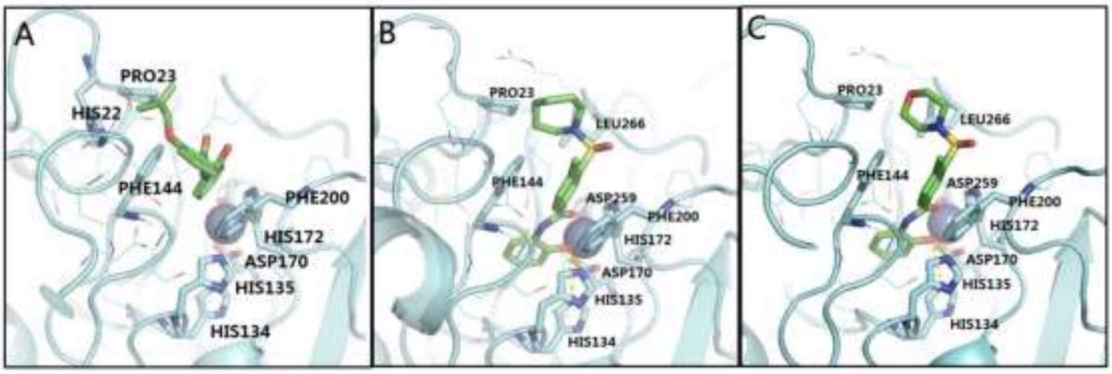

Figure S3. The predicted binding modes of three inactive compounds, i.e. 2-3 (A), 2-4 (B) and 2-5 (C). Color codes: light blue, HDAC3; green, hit compounds; blue sphere, zinc ion.

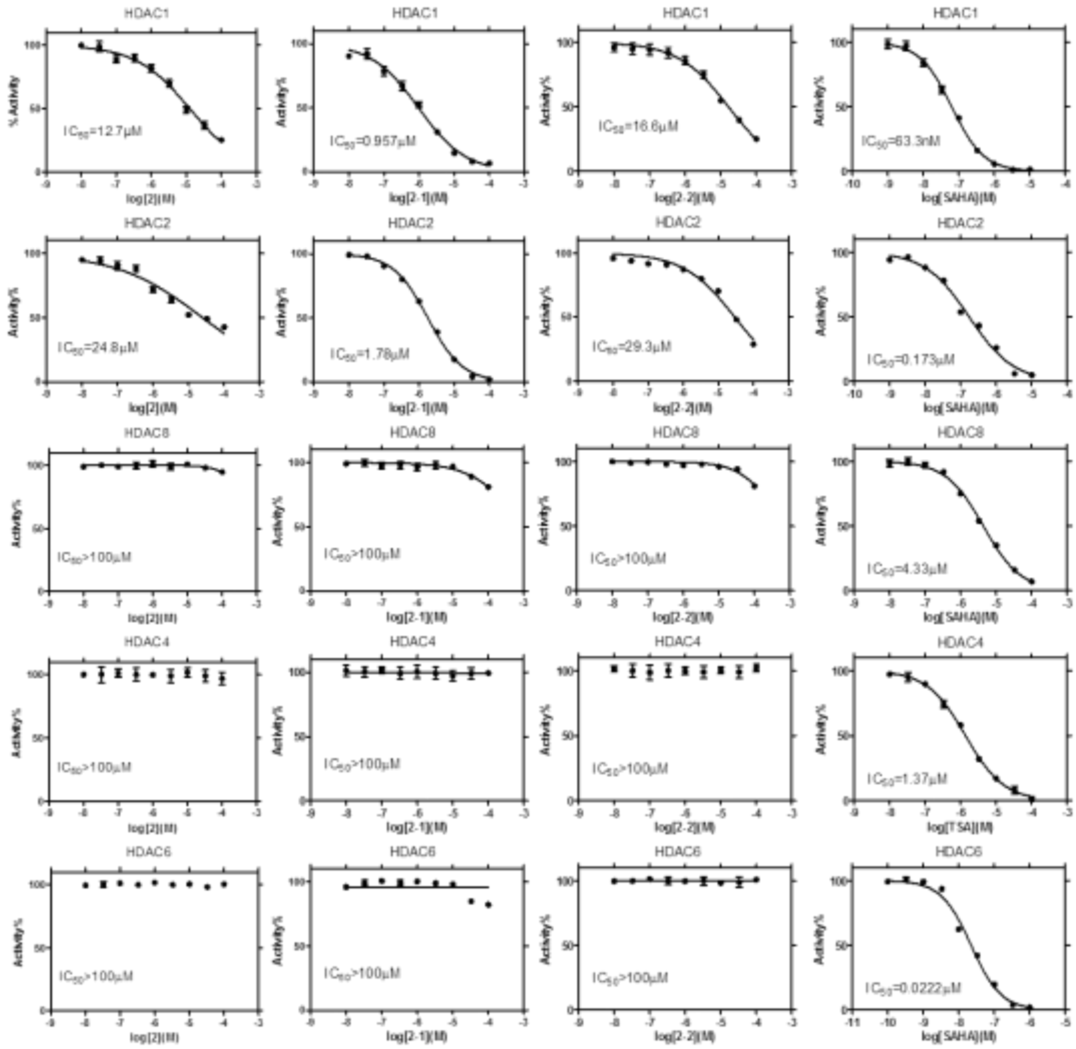

Figure S4. Dose response curves of each hit compound (i.e. 2, 2-1 and 2-2) for HDAC1, HDAC2, HDAC8, HDAC4 and HDAC6. SAHA or TSA (for HDAC4) was used as the positive control.
